# Opposite GC skews at the 5' and 3' ends of genes in unicellular fungi

**DOI:** 10.1186/1471-2164-12-638

**Published:** 2011-12-30

**Authors:** Malcolm A McLean, Itay Tirosh

**Affiliations:** 1 Department of Molecular Genetics, Weizmann Institute of Science, Rehovot, Israel

## Abstract

**Background:**

GC-skews have previously been linked to transcription in some eukaryotes. They have been associated with transcription start sites, with the coding strand G-biased in mammals and C-biased in fungi and invertebrates.

**Results:**

We show a consistent and highly significant pattern of GC-skew within genes of almost all unicellular fungi. The pattern of GC-skew is asymmetrical: the coding strand of genes is typically C-biased at the 5' ends but G-biased at the 3' ends, with intermediate skews at the middle of genes. Thus, the initiation, elongation, and termination phases of transcription are associated with different skews. This pattern influences the encoded proteins by generating differential usage of amino acids at the 5' and 3' ends of genes. These biases also affect fourfold-degenerate positions and extend into promoters and 3' UTRs, indicating that skews cannot be accounted by selection for protein function or translation.

**Conclusions:**

We propose two explanations, the mutational pressure hypothesis, and the adaptive hypothesis. The mutational pressure hypothesis is that different co-factors bind to RNA pol II at different phases of transcription, producing different mutational regimes. The adaptive hypothesis is that cytidine triphosphate deficiency may lead to C-avoidance at the 3' ends of transcripts to control the flow of RNA pol II molecules and reduce their frequency of collisions.

## Background

GC and AT skews are departures from Chargaff 's [1] second parity rule, that the amount of G (Guanine) equals the amount of C (Cytosine) in a single strand. Skews have been extensively studied in bacterial, mitochondrial and viral genomes, where they generally indicate origins of replication, with the leading strand tending to be G-biased and T-biased [2]. Skews received less attention in eukaryotic genomes although some studies reported skews in mitochondria [3], at origins of replication [4,5] and at transcription start sites in animals [6,7], plants [8], and fungi [9].

Interestingly, previous studies point to extensive variability in the presence and the exact patterns of skew among eukaryotes. For example, transcription start sites are typically G-biased in mammals but C-biased in invertebrates [6], and analysis of fungal species identified significant skews only among five of the seven species examined [9]. This variability may indicate that the skews themselves encode some regulatory information (the selective hypothesis) or, alternatively, that the skews are a byproduct of mutational pressure (the mutational hypothesis). This latter possibility would imply that different mutational mechanisms are operating in different species. These hypotheses are not mutually exclusive, and both imply much broader implications.

Previous studies hypothesized that GC and AT skews in genes, and particularly at transcription start sites, are caused by transcription-coupled mutations [10]. As the two strands of DNA are separated by the RNA polymerase II complex, each strand is placed in a different environment, causing different mutation pressures and thus strand asymmetries. For example, deamination of cytosine to thymine (C- > T) has been proposed to preferentially occur at the coding strand [11-13]. Notably, skews are found at most genes in single-celled organisms and in plants, which do not segregate their germ lines, but are only found at genes which are expressed in the germ cells of animals, consistent with the transcription-coupled mutation hypothesis [14-16].

Here we report a consistent pattern of GC skew across most unicellular fungi, while the model budding and fission yeasts are among the few exceptions examined. Surprisingly, we show that the 5'-ends of genes are C-biased but that the skew changes signs and is G-biased towards the 3'-ends of genes. We propose two possible explanations, one concerning mutational pressure, and one adaptive.

## Results

### Asymmetrical pattern of GC-skew within genes of most unicellular fungi

**Figure 1:**
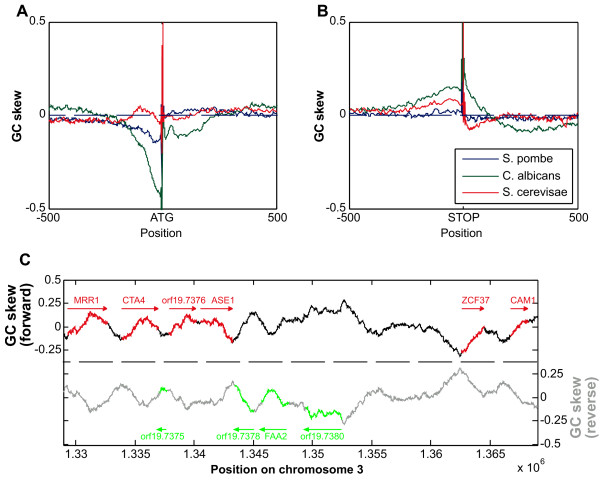
**GC skews around ATG and stop codon in three fungal species**. All genes are superposed to the ATG (a) or stop codon (b), and total skew for each position calculated. GC skew at the G of the ATG is 1.0 by definition, as is GC skew at the G of the stop codon. GC skews in the coding region have been averaged over the three bases, and data has been smoothed slightly for clarity. *Candida albicans *skews are substantially larger than for the other species. (c) GC skew for a selected region of chromosome 3 in C. albicans, with a window size of 2000 nucleotides. Many but not all genes show a pattern of increasing GC skew in the direction of transcription.

We first examined the average pattern of GC-skew at three model yeast species, *Saccharomyces cerevisiae, Schizosaccharomyces pombe *and *Candida albicans*. In each species, we averaged the skews of all genes at each position (base pair) relative to the translation start and stop sites (Figure 1a, b). In all three species, we observe a trivial positive skew at the exact start and stop sites (ATG and stop codons are G-skewed). In addition, we find a tendency for negative GC-skew (preference for C) around the translation start sites and positive GC-skew (preference for G) around translation stop sites. Notably, the magnitude of these skews varies between species, with weak GC skews in *S. cerevisiae *and *S. pombe *and a much stronger GC skew in *C. albicans*. In fact, in *C. albicans*, simple inspection of the GC skew in relation to genes shows that many, but not all, genes have a pattern of GC skew that increases from 5' to 3' end. (Figure 1c)

To evaluate the scope of this pattern we examined for each gene whether the change in skew between the translation start and stop sites is significant. Genes were shuffled 1000 times, and GC skew in the 5' and 3' hundred base pairs calculated. If the difference in GC skew was in the top 5% of randomized data, we scored the GC skew change as significant. We found a significant change of GC skew along 33% of the genes in *C. albicans *but only 14% and 7.5% of the genes in *S. cerevisiae *and *S. pombe*, respectively. Thus, the observed pattern of GC skew is a common property of *C. albicans *genes but is much less frequent in the other model yeast species.

**Figure 2:**
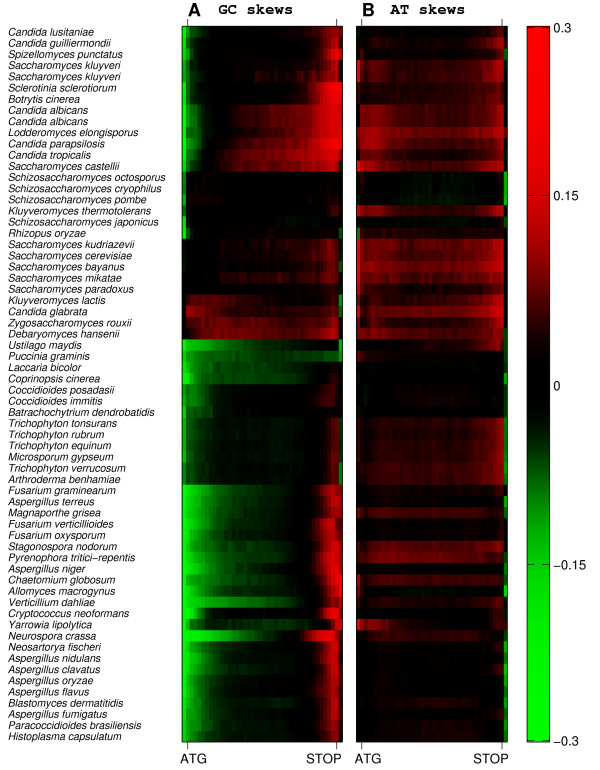
**GC (A) and AT (B) skews across genes in unicellular fungi**. Open reading frames are divided into 40 bins from the ATG to the stop codon, data for each species is pooled and the GC and AT skew calculated for each bin. The 200 bp upstream and downstream are also included. In almost all species we see a clear pattern of C-bias in the start of the gene changing sign to a G-bias towards the end. AT skews are flat but almost always positive.

The variability among the three model yeast species prompted us to examine the patterns of GC-skews among additional species. We thus downloaded the genomic sequences and gene annotations of all sequenced unicellular fungi and examined the average pattern of GC-skew in each species (Figure 2a). Surprisingly, we found that most species have a considerable average GC skew, as in *C. albicans *but not as in *S. cerevisiae *and *S. pombe*. Furthermore, most species follow the trend described above, with a relative preference for C at the 5'-end and for G at the 3'-end, suggesting that this is a conserved phenomenon among unicellular fungi.

The species can be divided roughly into four groups. At the top of Figure 2a can be seen those with a steep gradient of C-bias from the promoter to the begining of the coding region, followed by the majority of the gene G-biased. These are mainly *Candida *and *Saccharomyces *species. The second group is the *Schizosaccharomyces *species, which are C-biased in the promoter, but not GC-skewed within the gene, except to a very small extent. The third group is the *sensu stricto Saccharomyces *species, with a few non-*Saccharomyces *members. This group is not GC-skewed in the promoter, but shows a slight pattern of increasing G-bias in the gene, which does not extend into the 3' UTR. The fourth group, which consists of most of the species analysed, is C-biased for most of the gene, with a small G-biased region at the 3' end, which often extends into the UTR. As expected, closely-related species tend to share the same GC-skew signature, although there are some exceptions. For example, *Candida glabrata *and *Saccharomyces castellii *are closely related (Despite its genus name, the *Candida glabrata *genome is more similar to the genomes of *Saccharomyces *than to those of Candida species), but *S. castellii *is clearly in group one and *C. glabrata *in group three.

### GC-skews affect amino-acid choice

**Figure 3:**
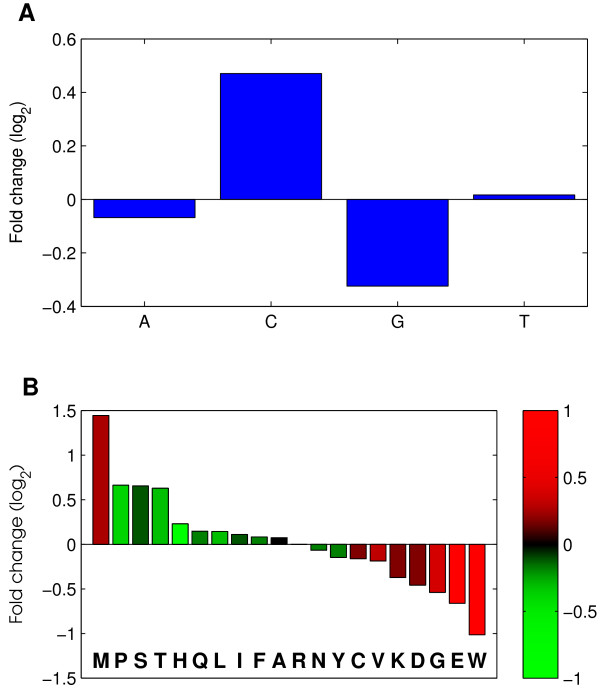
**Nucleotide and amino acid frequency ratios between the 5'- and 3'-ends of genes.** The 21 amino acids from the 5'-ends and 20 amino acids from the 3'-ends of all C. albicans genes (and the corresponding nucleotides) were compared by calculating the 5'/3' frequency ratio of each nucleotide (a) or amino acid (b). Bars in (b) are colored by the average GC-skew of the codons that encode for the corresponding amino acid (see colorbar), demonstrating that negatively GC-skewed amino acids are enriched at the 5'-ends of genes while positively GC-skewed amino acids are enriched at the 3'-ends of genes; Methionine is an exception because it is the first amino acid of all genes.

Since the pattern of GC skew shifts from negative to positive along genes, we examined the abundance ratio of each nucleotide, between the 5' and 3' ends. Strikingly, A and T showed only minor deviations in abundance between 5' and 3' ends, whilst G and C showed considerable gene-position effects, with G being highest at the 3' end, and C highest at the 5' end. (Figure 3a).

Next we examined if the usage of amino acids also differs between 5'-ends and 3'-ends. Indeed, we find that most amino acids are biased to either 5'-ends or 3'-ends of *C. albicans *genes, in agreement with the GC-skew of the corresponding codons (Figure 3b). (*P *< 0.001 for all amino acids except A, C, F, N, and R; Chi-square test). For example, the Tryptophan (W) codon, UGG, is G-biased, and tryptophan is approximately twice as common at the 3'-ends than at the 5'-ends of *C. albicans *genes.

The correlation between GC-skew and a preference for certain amino-acids could indicate that the choice of amino acids is under selection and that this generates the observed pattern of GC skew. For example, we noted that pairs of similar residues (serine and threonine, leucine and isoleucine, aspartic acid and glutamic acid) often have similar fold-change between the 5' and 3' ends (Figure 3b). However, the pattern of increased GC skew from the 5'-end to the 3'-end continues beyond the coding region, both into promoters and into 3'- UTRs (Figures 1, 2). In fact, for most species, the strongest (negative) GC skew is observed at the promoter and then decreases sharply at the coding region. Thus, the patterns of GC-skew could not be accounted for solely by selection for amino-acid choice, and we propose that it primarily reflects mutational load.

The mutational load hypothesis would predict a greater effect at fourfold-degenerate positions, as these positions are believed to be less constrained, yet we find an effect of similar magnitude at non-degenerate positions (i.e. first and second codon positions) (additional file 1, **Figure S1**). This could, in principle, indicate a role for selection for amino-acid choice in generating the observed patterns of GC-skew. However, we note that fourfold-degenerate positions are constrained by the choice of codons, as some codons are more efficiently translated than others. Notably, efficient codons are particularly enriched with Cytosines (over Guanines) in their third-base positions (additional file 1, **Figure S2**), and are depleted the 5' ends of genes [17,18]. This should generate an opposite bias to the one that we observe, with abundance of Cytosines (negative GC-skew) at the 3' ends of genes. We thus speculate that selection pressure for increased translational efficiency along genes [17,18] reduces the GC-skew at fourfold-degenerate positions. Accordingly, the effects of constraint for amino acid choice, at non-degenerate positions, and translational efficiency, at degenerate positions, could reduce the impact of mutational load to a similar extent, thereby generating seemingly codon-position independent increase in GC-skews along genes.

### Uniform AT-skews are correlated with positive, but not negative, GC-skews

**Figure 4:**
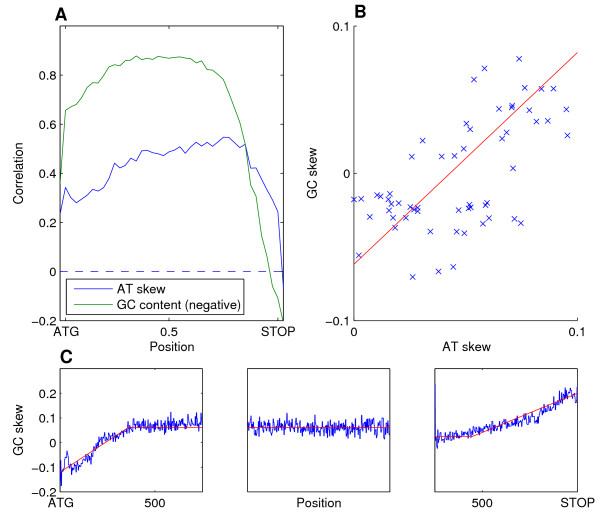
**Correlation between AT skew and GC skew**. (a) Pearson correlation between local GC skew and either AT skew (blue) or GC content (green) averaged over the entire coding regions. (b) Overall AT skew plotted against overall GC skew for 64 species, R = 0.52. (c) Average pattern of GC-skews over all *C. albicans *genes at three regions (5', middle and 3'), along with linear fits.

Most species also have considerable AT-skews within genes, although somewhat lower than GC-skews (Figure 2b), and the extent of AT and GC skews are correlated both across genes within the same species (Figure 4a) and across different species (Figure 4b). However, in contrast to GC-skews, AT-skews are largely constant across the length of genes, with a general preference for A over T (positive AT-skew) and correlate primarily with the extent of GC-skew at the middle of genes, but less so at the 5' and 3' ends (Figure 4a). These results suggest that a common mechanism generates a uniform and positive AT- and GC-skew throughout genes, but that additional unknown mechanisms generate the decreased GC skew at 5' ends and increased GC skew at 3'-ends. Consistent with this possibility, we note that for some species such as *C. albicans*, the pattern of skew fits well with a model of three regimes: a constant GC-skew at most of the gene, a gradual (linear) decrease of GC-skew at the 5'-end and a gradual (linear) increase at the 3'-end (Figure 4c).

### GC-skews may be associated with transcription and DNA methylation

The observation that GC-skews extend from coding region to the surrounding UTRs and is in fact strongest at the 5'-UTR supports the possibility that GC-skews are generated by a mechanism that is coupled to transcription. To further examine this possibility we examined the correlation between GC-skew and expression levels across *C. albicans *genes. We found significant (*P *< 0.001), yet very weak correlations with both the negative GC-skew at the 5'-UTR (*R *= -0.096) and the positive GC-skew at the 3'-UTR (R = 0.071) (additional file 1, **Figure. S3**). These weak correlations support the possibility of transcription-coupled GC-skews but suggest that the extent of GC skew is not determined primarily by the level of transcription but rather largely by other aspects of the transcriptional process. For example, PolII dynamics (e.g. stalling) and the gene-specific binding of various regulatory factors to the transcriptional machinery may have strong effects on the degree of GC-skew.

Methylation of cytosine bases is an evolutionary ancient system that has been lost separately in *S. cervisiae *and *S. pombe *(which have weak skews), but retained in *N. crassa *and *C. albicans *(which have strong skews) [19,20], and therefore might be related to the mechanisms that generate the observed GC-skews. However, we did not detect any significant difference in GC skews between the 150 methylated *Candida *genes listed in [21] and the genome-wide level of GC skew, nor did we find any correlation between the level of methylation in those 150 genes and their GC skews (data not shown).

## Discussion

### Mutational pressures may differ between the 5' and 3' ends of genes

GC-skews within genes have been described by multiple previous studies and have been traditionally explained by transcription-coupled mutational pressures [13,22-24]. The tripartite nature of the GC skew that we observe across dozens of unicellular fungi (i.e. C biased at the 5' end, intermediate at a central region, and G-biased at the 3' end), is not consistent with a uniform mutational pressure and may thus suggest that mutational pressures vary within genes. Such intragenic variability could be related to the various auxiliary proteins and co-factors that bind transiently to RNA Pol II during initiation, elongation, or termination, and may generate different regimes of mutations or DNA repair. For example, PolII undergoes frequent abortive initiation and is often stalled at the 5'-ends of genes, while bound by various co-activators [25]. This may define a distinct transcription initiation state at the genes 5'-ends that could facilitate certain mutation or repair mechanisms, thereby accounting for our observation that GC-skew is typically most negative around the TSS and then gradually increases within the genes. Similarly, transcription termination involves the binding of multiple accessory proteins which may affect the pattern of mutations and repair at the 3'-end. However, in contrast to the 5'-end, the positive GC-skew at the 3-end is strongest at the stop codons rather than the transcription termination region. This seems inconsistent with an influence of transcription termination, unless termination factors bind to Pol II before termination and around the position of the stop codon.

DNA methylation has been lost in *S. cervisiae *and *S. pombe*, but retained in *C. albicans *and *N. crassa*. This pattern is consistent with a role for methylation in generating GC-skews in fungi, although it is based only on four species while (to the best of our knowledge) the presence or absence of DNA methylation is not known in the other unicellular fungi. It has recently been shown that methylated regions of DNA show a greater rate of C to T deamination in *C. albicans *[21]. This could generate skew if the methylation was itself strand-biased. However, we did not find any relationship between GC skew and the presence or the extent of DNA methylation in *C. albicans *[21].

C-to-T deamination has been proposed as a dominant source of GC-skews and as mentioned above is also linked to DNA methylation. However, C-to-T deamination both increases GC-skew (by removing a C) and decreases AT-skew (by adding a T) and thus would generate inversely correlated patterns of GC and AT-skews. The observation that GC- and AT-skews are positively (rather than negatively) correlated, and the relative uniformity of AT skews across genes argues against a major role of C-to-T deamination in generating the observed patterns of GC and AT skews and instead suggests that these patterns reflect other types of strand-biased mutations. There is selective pressure on AT skew because T-rich codons tend to code for more expensive amino acids [26]. We also note that GC skew is negatively correlated with GC content (Figure 4a), perhaps implicating base composition in influencing patterns of mutation and repair. Interestingly, nucleotide excision repair acts preferentially on the transcribed strand and could thus promote transcription-associated GC and AT skews, as recently suggested from analysis of cancer genomes [27,28].

### Patterns of skew may reflect an adaptive tendency for C-avoidance at 5' ends

In contrast to the mutational pressure hypothesis we can also consider an adaptive explanation. According to this possibility GC-skew influences the rate of transcription elongation (see below) and, as recently suggested for translation elongation [17,18], it might be beneficial to have initially slow elongation (at the 5'-end) followed by faster elongation (at the 3-'end) as this reduces the degree of collisions between RNA polymerases [29] (or ribosomes). This argument relies on the observation that CTP is found at a lower abundance than ATP, GTP and UTP: A relative depletion of CTPs was previously observed in multiple organisms including mammalian cytosols (~70% more GTPs than CTPs) [30], chick fibroblasts [31] and plants [32]. A significant, albeit weaker, CTP depletion was also observed in *S. cerevisiae *(~36% more GTPs than CTPs) [33]. CTP depletion was also proposed to generate a global C-avoidance across various bacterial genomes [34]. Thus, a high C-frequency at the 5' ends (i.e. negative GC-skew) may slow down the initial rate of elongation if CTP loading is a rate limiting step for elongation under physiological conditions. Elongation rate may then increase along the gene as the C-frequency decreases, and this strategy may decrease the extent of PolII collisions. This hypothesis raises two predictions that could be tested in the future: (i) CTP pools are lower in GC-skewed than non-skewed species, and (ii) the kinetics of RNA elongation is relatively slow for C-rich transcripts at physiological NTP concentrations.

It is tempting to further speculate why this composite GC-skew is absent in other organisms based on the collision avoidance hypothesis. First, the model budding and fission yeasts were chosen as laboratory model organisms partially since they can grow easily on minimal media. This could indicate that they synthesize most of their essential molecules (including CTP) rather than scavenging it from the environment and thus have higher CTP abundance. Second, higher eukaryotes have much longer, intron-rich genes, and therefore may rely on other mechanisms for avoiding RNA Pol II collisions.

## Conclusions

Most unicellular fungi have significant GC-skews in transcribed sequences, with a negative skew at the 5'-end and a positive skew at the 3'-end. This pattern of skew influences amino acid composition at the ends of proteins, but cannot be accounted by selection for amino acid composition. We propose two models that could explain this pattern: mutational pressures that differ between the 5' and 3'-ends of genes, for example due to preferential nucleotide excision repair at 5' ends, or alternatively, selection for avoiding Pol II collisions through preferential C-avoidance at the 3' ends of genes.

## Methods

GC skew is calculated as (G - C)/(G + C) and AT skew as (A - T)/(A + T), giving a symmetrical metric from -1 to 1.0. It is possible to calculate either using a sliding window, which raises the issue of the correct window length, or by superposing all genes to the ATG or stop codon, and calculating the skew at each position. If the superposition method is used then the skew can be seen in perfect base pair resolution, at the cost of losing differences between genes and chromosome position effects.

The superposition method shows the phenomenon to better effect, and was used to generate Figure 1. However it does not show as well how skew changes across the gene. Gene sequences for unicellular fungi were downloaded from public repositories. Each open reading frame was divided into forty sections. Genes were then combined to produce twenty average GC skews across the gene for each organism. GC skews for the 200 basepairs upstream and downstream of the open reading frame were also calculated, to produce Figure 2. The same process was used for AT skews.

The N-terminal 21 residues and C-terminal 20 residues from all protein-coding genes in C. albicans were taken to see how amino acid choice was affected by the GC skew. Amino acids were ranked by fold change and coloured by codon GC skew [Figure 3].

Absolute gene mRNA expression levels were taken from [35] and the Pearson correlation between expression and GC skew in the 75 bases at the 5' end and the 75 bases at the 3' end 3 calculated.

Methylation data was taken from [21] and the Pearson correlation between the GC skews in the 75 bases at the 5' end and the 75 bases at the 3' end and level of methylation calculated for the 150 methylated genes listed. A 2 sample t-test was performed to test whether the mean GC skew of the 150 methylated genes was greater than expected by chance.

A three line model describes the GC skews of most sufficiently large sets of genes reasonably well. The model was fit to the data by dividing data into three parts: a 750 bp 5' region aligned to the ATG, a 750 bp 3' region aligned to the STOP, and a 750 bp region aligned to the centre of genes. A three line model can be described by three parameters; a: distance from GC skew at ATG to GC skew at centre, b: end of 5' ramp, in base pairs, c: the GC skew of the central section, d: start of 3' ramp, in base pairs from STOP, e: distance from GC skew at STOP to GC skew at centre. Parameter c can be fit simply by taking the average GC skew of the central section of data. The other parameters were then fit by exhaustive enumeration to minimize squared error. to produce Figure 4c.

### Data sets

Data sets were downloaded as lists of genes or open-reading frames from public resource servers. For most species, genes are assigned by automatic algorithms.

#### From the Broad Institute

Allomyces macrogynus, Arthroderma benhamiae, Aspergillus clavatus, Aspergillus flavus, Aspergillus fumigatus, Aspergillus nidulans, Aspergillus niger, Aspergillus oryzae, Aspergillus terreus, Batrachochytrium dendrobatidis, Blastomyces dermatitidis, Botrytis cinerea, Candida albicans, Candida albicans, Candida guilliermondii, Candida lusitaniae, Candida parapsilosis, Candida tropicalis, Chaetomium globosum, Coccidioides immitis, Coccidioides posadasii, Coprinopsis cinerea, Cryptococcus neoformans, Debaryomyces hansenii, Fusarium graminearum, Fusarium oxysporum, Fusarium verticillioides, Histoplasma capsulatum, Laccaria bicolor, Lodderomyces elongisporus, Magnaporthe grisea, Microsporum gypseum, Neosartorya fischeri, Neurospora crassa, Paracoccidioides brasiliensis, Puccinia graminis, Pyrenophora tritici-repentis, Rhizopus oryzae, Schizosaccharomyces cryophilus, Schizosaccharomyces japonicus, Schizosaccharomyces octosporus, Schizosaccharomyces pombe, Sclerotinia sclerotiorum, Spizellomyces punctatus, Stagonospora nodorum, Trichophyton equinum, Trichophyton rubrum, Trichophyton tonsurans, Trichophyton verrucosum, Ustilago maydis, Verticillium dahliae

#### From the Saccharomyces Genome Database (SGD)

Saccharomyces bayanus, Saccharomyces castellii, Saccharomyces cerevisiae, Saccharomyces kluyveri, Saccharomyces kudriazevii, Saccharomyces mikatae, Saccharomyces paradoxus

#### From the Pasteur Institute

Candida_glabrata, Kluyveromyces_lactis, Kluyveromyces thermotolerans, Saccharomyces_kluyveri, Yarrowia_lipolytica, Zygosaccharomyces_rouxii

#### Expression data *for C. albicans*

Downloaded as supplemental data from Bruno (2010) Comprehensive annotation of the transcriptome of the human fungal pathogen *Candida albicans *using RNA-seq [35].

#### Methylation data for *C. albicans*

Downloaded as supplemental data from Mishra *et al *(2011) DNA Methylation regulates phenotype-dependent transcriptional activity in *Candida alibicans *[21]

## Authors' contributions

MAM conceived the study, and wrote the computer programs to analyse data and produce figures. IT suggested analyses and discussed the interpretation of results. Both authors wrote the paper and agreed on conclusions. Both authors have read and approved the final manuscript.

## Supplementary Material

Additional file 1 **Supplementary figures. Figure S1 - GC skews by position with gene by codon base**. GC skews vary by position within the codon. **Figure S2 - GC skews of third base degenerate positions, in low and high expression genes**. GC skews at the third base degenerate position calculated for genes with 10% lowest and 10% highest expression. **Figure S3 - Correlation between GC skew and expression**. Data density plots of GC skews versus expression levels. Click here for file
