# Supplementary material for: Opposite GC skews at the 5' and 3' ends of genes in unicellular fungi
Source: BMC Genomics. 2011 Dec 30;12:638. doi: 10.1186/1471-2164-12-638 (PMC3315797; doi:10.1186/1471-2164-12-638)
Supplement: Additional file 1 — Supplementary figures. Figure S1 - GC skews by position with gene by codon base. GC skews vary by position within the codon. Figure S2 - GC skews of third base degenerate positions, in low and high expression genes. GC skews at the third base degenerate position calculated for genes with 10% lowest and 10% highest expression. Figure S3 - Correlation between GC skew and expression. Data density plots of GC skews versus expression levels. [file 1471-2164-12-638-S1.DOC]

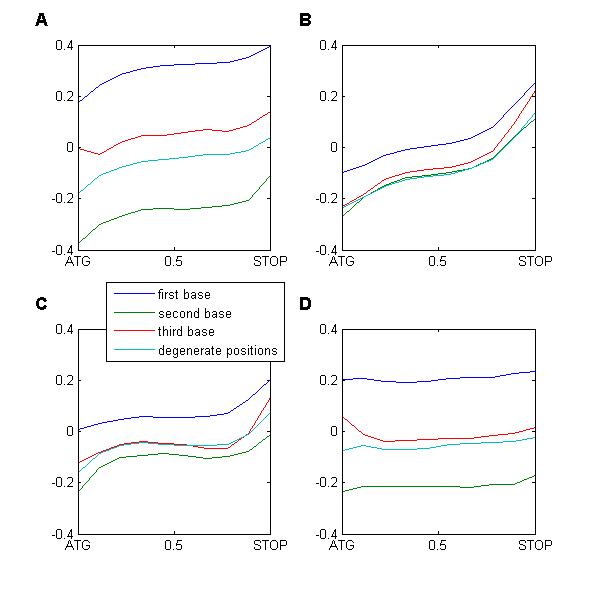


**Figure S1.** GC skews by position along the gene and codon base in *Candida albicans* (A), *Neurospora crassa* (B), *Aspergillus nidulans* (C) and the non-GC skewed species *Schizosaccharmoyces pombe* (D), First base positions are more G-biased than second base positions, because of amino acid frequencies, but the lines are nearly parallel, indicating an identical contribution to the pattern of skew. Third base positions are also nearly parallel, as are third base degenerate positions. Since third base degenerate positions are subject to less selective pressure, this is surprising.


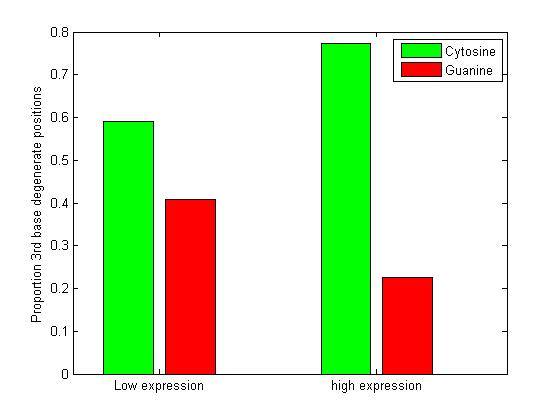


**Figure S2.** We took the 10% genes with lowest expression (and thus inefficient codons) and 10% of genes with highest expression in *Candida albicans* (and thus efficient codons), and compared the frequencies of Guanine and Cytosine at fourfold degenerate positions. C is dramatically increased over G in the highly-expressed group, indicating that efficiently translated codons are more likely to contain a C at fourfold degenerate position.


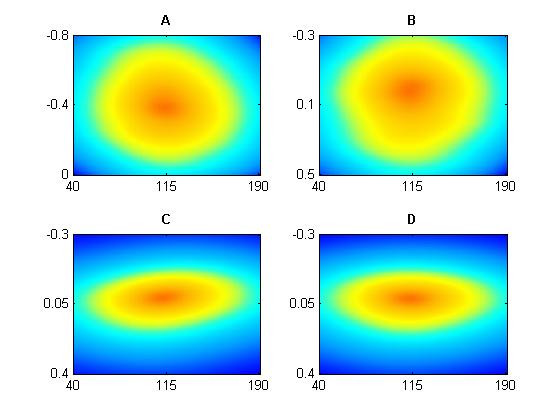


**Figure S3**. Data density plots of GC skew versus expression, in *Candida albicans*. Expression levels are from [1]. Data density is calculated as sum of points, weighted by reciprocal squared distance from the pixel, and shown as heat map. N is approximately 6,000 Plots show expression (X axis, arbitrary scale) versus (A) GC skew at 5' UTRs, R = -0.096416 (B) 3' UTRS, R = 0.070715 , (C) GC skew of the whole genes, R = 0.197959) (D) plot C randomized, R = 0.004370. In all cases except the randomized data, p is less than 10^-6^. Whilst the data passes statistical tests for correlation, the effect size is very small.

1. Bruno VM, Wang Z, Marjani S, Euskirchen GM, Martin J, Sherlock G, Snyder M: **Comprehensive annotation of the transcriptome of the human fungal pathogen Candida albicans using RNA-seq** *Genome Research* 2010, **20**:1451-1458.
